# Identification and Molecular Characterization of Four New *Blastocystis* Subtypes Designated ST35-ST38

**DOI:** 10.3390/microorganisms11010046

**Published:** 2022-12-23

**Authors:** Jenny G. Maloney, Aleksey Molokin, Raimundo Seguí, Pablo Maravilla, Fernando Martínez-Hernández, Guiehdani Villalobos, Anastasios D. Tsaousis, Eleni Gentekaki, Carla Muñoz-Antolí, Debora R. Klisiowicz, Camila Y. Oishi, Rafael Toledo, J. Guillermo Esteban, Pamela C. Köster, Aida de Lucio, Alejandro Dashti, Begoña Bailo, Rafael Calero-Bernal, David González-Barrio, David Carmena, Mónica Santín

**Affiliations:** 1 Environmental Microbial and Food Safety Laboratory, Agricultural Research Service, United States Department of Agriculture, Beltsville, MD 20705, USA; 2 Department of Pharmacy, Pharmaceutical Technology and Parasitology, Parasitology Area, Faculty of Pharmacy, Valencia University, Avda. Vicent Andrés Estellés s/n, 46100 Burjassot, Valencia, Spain; 3 Global Omnium, Gran Via del Marqués del Túria 19, 46005 Valencia, Valencia, Spain; 4 Departamento de Ecologia de Agentes Patogenos, Hospital General Dr. Manuel Gea Gonzalez, Mexico City 14080, Mexico; 5 Laboratory of Molecular and Evolutionary Parasitology, RAPID Group, School of Biosciences, University of Kent, Canterbury CT2 7NJ, UK; 6 School of Science, Mae Fah Luang University, Chiang Rai 57100, Thailand; 7 Department of Basic Pathology, Biological Sciences Area, Paraná Federal University, Av. Cel. Francisco H. dos Santos 100, Curitiba 19031, Brazil; 8 Parasitology Reference and Research Laboratory, Spanish National Centre for Microbiology, Majadahonda, 28222 Madrid, Spain; 9 SALUVET, Department of Animal Health, Faculty of Veterinary, Complutense University of Madrid, 28040 Madrid, Spain; 10 Center for Biomedical Research Network in Infectious Diseases (CIBERINFEC), Health Institute Carlos III, 28222 Madrid, Spain

**Keywords:** *Blastocystis*, *B. lapemi*, subtype, Oxford Nanopore MinION, *ssu* rRNA

## Abstract

Three recent studies of *Blastocystis* epidemiology in mammalian hosts identified four novel sequences that appeared to share *B. lapemi* as the most similar sequence. However, full-length *ssu* rRNA gene sequences were not available to confirm the validity of these new subtypes. In the present study, Nanopore MinION sequencing was used to obtain full-length reference sequences for each of the new subtypes. Additionally, phylogenetic analyses and pairwise distance comparisons were performed to confirm the validity of each of these new subtypes. We propose that the novel sequences described in this study should be assigned the subtype designations ST35-ST38. The full-length reference sequences of ST35-ST38 will assist in accurate sequence descriptions in future studies of *Blastocystis* epidemiology and subtype diversity.

## 1. Introduction

*Blastocystis*, a member of the stramenopiles, is a ubiquitous intestinal microorganism that infects/colonizes a broad range of human and non-human hosts. Indeed, *Blastocystis* is regarded as one of the most common microeukaryotes present in the human gastrointestinal tract [1]. Like other enteric protists, transmission is thought to be primarily via the faecal oral route, either directly through contact with infected humans or animals, or indirectly through the ingestion of water or food that is contaminated with faecal material from infected hosts [2].

The clinical significance of *Blastocystis* infection/colonization is not fully understood. Asymptomatic carriage seems to be the most common presentation of this microorganism. However, human infections have been associated with gastrointestinal illnesses, including diarrhoea and irritable bowel syndrome, or even extraintestinal manifestations, such as urticaria and other skin disorders [3,4,5]. Whether the factors that determine the outcome of the infection are host or parasite dependent remains to be defined, as no clear links between the presence of *Blastocystis* and pathogenicity have been firmly established. Evidence from recent metagenomic studies suggests that *Blastocystis* may be part of the healthy gut microbiota in most circumstances [6,7,8].

A great deal of genetic diversity is contained within the *Blastocystis* species complex. Currently a subtyping system, which is based on sequence analyses of the small-subunit ribosomal RNA (*ssu* rRNA) gene, is used to differentiate between sequence variants of *Blastocystis* [1,9]. However, the subtyping system only applies to isolates obtained from mammalian and avian species. Using this system, at least 30 subtypes (ST1–ST17, ST21, and ST23–ST34) have been proposed and validated [1,10,11]. Of the 14 subtypes, which have been reported in humans, ST1 to ST4 are the most common, while reports of ST5-ST10, ST12, ST14, ST16, and ST23 in humans range from relatively uncommon to rare [12,13,14,15,16,17]. To our knowledge, all other *Blastocystis* subtypes have only been documented in non-human animal species and are currently considered to have a limited or negligible zoonotic potential [2]. Because of the apparent loose host specificity of multiple *Blastocystis* subtypes, surveys of *Blastocystis* prevalence and subtype diversity from a variety of hosts and geographic locations are of interest and important in clarifying the epidemiology and zoonotic potential of *Blastocystis* subtypes.

Three separate recent studies of *Blastocystis* subtype diversity from human or animal hosts produced sequence data which indicated the presence of four potentially novel subtypes. These studies aimed to describe *Blastocystis* subtype diversity in humans from Brazil [18], wild mammals from Mexico [19], and captive water voles from the UK [20]. Interestingly, all studies identified isolates that have *Blastocystis lapemi* as the most similar nucleotide sequence present in the GenBank database. The *B. lapemi* isolate was originally obtained from a sea snake of the genus *Lapemis* [21]. However, at the time of their original description, the data needed to confirm the status of these isolates as new subtypes were not available. The present study employed long-read sequencing using Oxford Nanopore Technology’s MinION to obtain the full-length *ssu* rRNA gene for four isolates to fulfil the requirements to validate and descriptive four novel *Blastocystis* isolates.

## 2. Materials and Methods

### 2.1. Sources and Descriptions of Samples

The samples used in this study came from three separate investigations of *Blastocystis* subtype diversity [18,19,20].

In the first study, the molecular characterization of *Blastocystis* isolates obtained from humans living in Paranaguá Bay, on the coast of the Paraná state in southern Brazil, identified two isolates, which differed by a single SNP at the barcoding region (*ca.* 600 bp), that have a *B. lapemi* sequence as the most similar (92%) sequence present in the GenBank database [18]. DNA for one of those isolates was available and it is referred to as 168 in the present study.

The second study aimed to investigate *Blastocystis* in wild animals from two sylvatic areas of Mexico [19]. Three isolates obtained from an opossum (*Philader opossum*) (Flo2), a little yellow-shouldered bat (*Sturnira lilium*) (MFlo44a), and a rodent (*Heteromyidae*) (FCP5) have a *B. lapemi* sequence as the most similar sequence present in the GenBank database (barcoding region) and clustered in separate and strongly supported clades that were close to *B. lapemi*. The nucleotide sequence from rodent isolate FCP5 shared a 91% identity with *B. lapemi* (AY266471). The opossum isolate Flo2 and bat isolate MFlo44a did not have differences between them and also shared 91% identity with *B. lapemi* (AY590115). In the present study, DNAs from a little yellow-shouldered bat (MFlo44a) and rodent (FCP5) were used.

The third study aimed to investigate the association of *Blastocystis* with other protist residents in the gut of captive European water voles (*Arvicola amphibius*) and was conducted in the UK; samples from this study were also used in microbiome studies in water voles [20,22]. Samples from two water voles (Q99 and Q52) were found at the barcoding region to have *B. lapemi* as the most similar sequence present in the GenBank database (91%). DNA from the water vole Q52 was used in the present study.

DNA from each sample was shipped to the Environmental Microbial and Food Safety Laboratory in Beltsville, MD, USA for further analysis.

### 2.2. PCR Amplification and Sequencing of the Full-Length ssu rRNA Gene

To obtain the full-length nucleotide sequence of the *ssu* rRNA gene of the novel genetic variants of *Blastocystis*, a previously described Nanopore sequencing strategy was used with the following updates [11]. Briefly, a PCR using the MinION-tailed primers forward (5′–TTT CTG TTG GTG CTG ATA TTG C AAC CTG GTT GAT CCT GCC AGT AGT C–3′) and reverse (5′–ACT TGC CTG TCG CTC TAT CTT C TGA TCC TTC TGC AGG TTC ACC TAC G–3′) (MinION adapter nucleotide sequences underlined), which amplify most eukaryotic organisms’ full-length *ssu* rRNA gene sequence, was performed using the high-fidelity proofreading polymerase contained in KAPA HiFi HotStart ReadyMix (KAPABioSystems, Cape Town, South Africa). Initial denaturation was performed at 98 °C for 5 min, followed by 35 cycles of amplification: 20 s at 98 °C, 45 s at 60 °C, and 90 s at 72 °C. The final extension continued for 5 min. PCR amplicons were purified using a 0.5X AMPure XP beads (Beckman Coulter, Brea, CA, USA) to sample ratio and quantified on a Qubit fluorometer (ThermoFisher Scientific, Waltham, MA, USA). To prepare the Nanopore sequencing libraries, the Oxford Nanopore Technologies (ONT) SQK-LSK110 Ligation Sequencing Kit and EXP-PBC001 PCR Barcoding Kit (ONT, Oxford, UK) were used following the manufacturer’s protocol for PCR Barcoding Amplicons (PBAC12_9112_v110_revB_10Nov2020) and loading guidelines for R10.3 flow cells. A modification to the barcoding PCR protocol included the use of the KAPA HiFi polymerase described above instead of the NEB LongAmp Taq, with the exception of a 62 °C annealing temperature that is specific to the nanopore primers. Final libraries were quantified and diluted to ensure 75 fmol in 12 µL was loaded onto an R10.3 flow cell (FLO-MIN111) for sequencing on an ONT MinION Mk1C.

### 2.3. Bioinformatics Analysis (Read Processing and Consensus Building)

Basecalling was performed using Guppy v4.4.1 and the high accuracy model using the flag -c dna_r10.3_450bps_hac.cfg with a minimum quality score cut off of 7 for filtering low quality reads. FASTQ reads were then length filtered to include only reads between 1700 and 2100 nucleotides. Read processing and consensus building were performed as previously described [11] with the following updated workflow. Briefly, reads were corrected using canu v2.1, followed by adapter trimming and the retrieval of reads containing intact forward and reverse primers. Reads were clustered using the VSEARCH --cluster_fast command (vsearch v2.15.1) at a 98% identity threshold, checked for chimeras, and filtered for off-target sequences [23]. Clusters were then polished with racon v1.4.20, clustered again, and polished once more with Medaka v1.4.3 using the model r103_min_high_g360. The nucleotide sequences generated were deposited in GenBank under the accession number OP720869-OP720872.

### 2.4. Phylogenetic Analyses

The full-length *ssu* rRNA gene nucleotide sequences obtained in this study, appropriate full-length *Blastocystis* reference nucleotide sequences obtained from the reference database found at http://entamoeba.lshtm.ac.uk/blastorefseqs.htm (accessed on 19 December 2022), as well as other full-length sequences available in GenBank, were included to generate a phylogenetic tree artificially rooted using *Proteromonas lacertae*, a stramenopile which is closely related to *Blastocystis*, as an outgroup. Nucleotide sequences were aligned with the Clustal W algorithm using MEGA X [24]. Phylogenetic analyses were performed using the neighbor-joining (NJ) method and pairwise distances were calculated with the Kimura 2-parameter model using MEGA X [24]. All ambiguous positions were removed for each sequence pair (pairwise deletion option). Bootstrapping with 1000 replicates was used to determine support for the clades generated.

## 3. Results

### 3.1. Full-Length ssu rRNA Gene Sequences

Full-length *ssu* rRNA gene sequences were obtained from all four samples included in the study, and all full-length sequences were nearly identical to the Sanger sequences which had been previously generated for these samples. The full-length sequence of 168 had only a single mismatched base 18 bases from the 3′ end of the Sanger sequence. Mflo44a had only one mismatch 10 bases from the 3′ end of the Sanger sequence. FCP5 had only one mismatch 11 bases from the 3′ end of the Sanger sequence. Q52 had only one mismatch 22 bases from the 3′ end of the Sanger sequence. Interestingly, the mismatch was represented by the same base in all sequences, with a C in the Sanger sequences being replaced with a T in the full-length sequences generated by Nanopore sequencing.

### 3.2. Phylogenetic Analyses

The four full-length *ssu* rRNA gene sequences from this study and reference sequences for all other accepted *Blastocystis* subtypes from avian and mammalian hosts (ST1–ST17, ST21, and ST23–ST34) were used to conduct phylogenetic analyses. Additionally, phylogenetic analyses, including full-length reference sequences which are available in GenBank for *Blastocystis* isolates from cold-blooded hosts (reptilian, amphibian, and insect), were conducted. All analyses included *P. lacertae* as an outgroup taxon to artificially root the trees.

**Figure 1:**
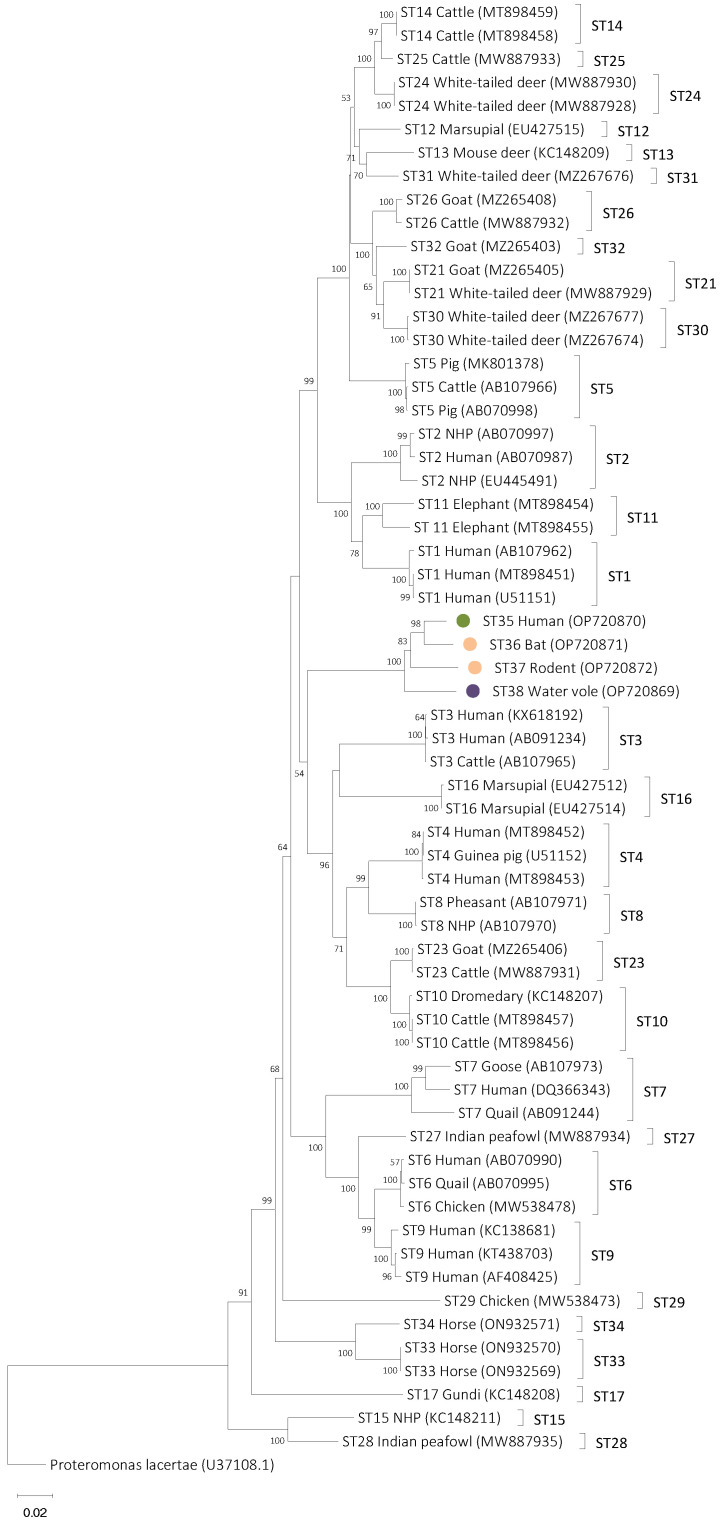
Phylogenetic relationships among the *Blastocystis* sequences generated in the present study (represented with coloured circles; in green Brazilian isolate 168, in purple UK isolate Q52, and in orange Mexican isolates MFlo44a and FCP5) and representative sequences of *Blastocystis* subtypes. Note that subtype designations are only assigned to isolates from mammalian and avian origin. The analysis was conducted by a neighbor-joining method. Genetic distances were calculated using the Kimura two-parameter model. This analysis involved 63 nucleotide sequences, and there were a total of 2141 positions in the final dataset. The percentage of replicate trees in which the associated taxa clustered together in the bootstrap test (1000 replicates) are shown next to the branches. Bootstrap values lower than 75% are not displayed. *Proteromonas lacertae* was used as an outgroup taxon to artificially root the tree. NHP means non-human primate.

**Figure 2:**
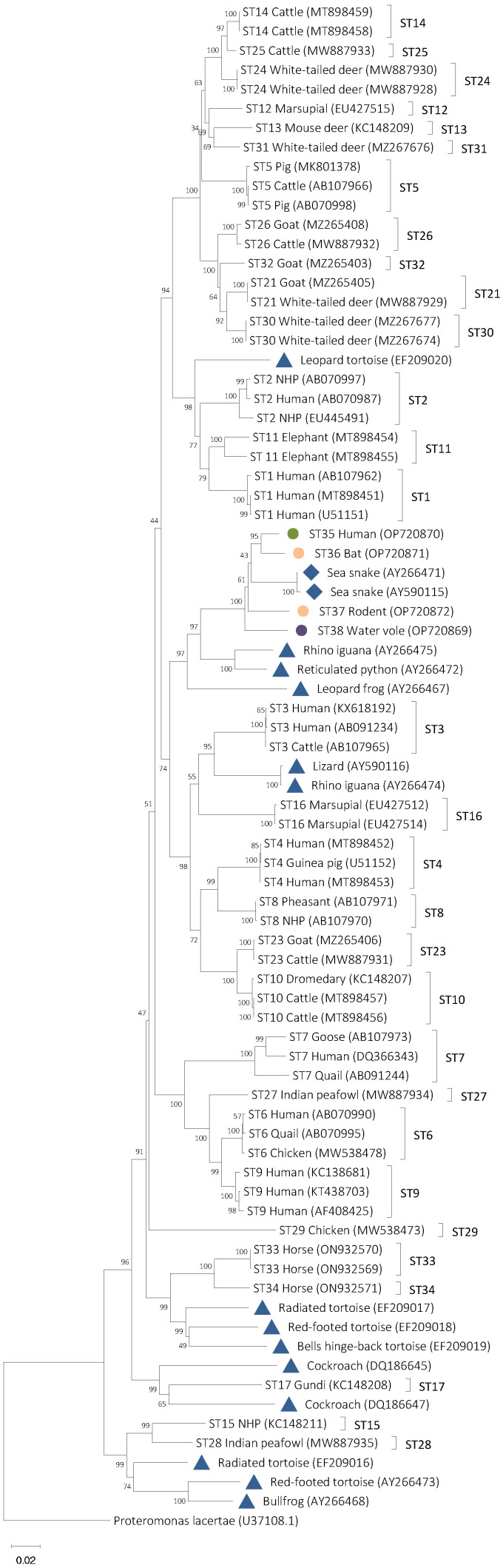
Phylogenetic relationships among the *Blastocystis* sequences generated in the present study (represented with coloured circles; in green Brazilian isolate 168, in purple UK isolate Q52, and in orange Mexican isolates MFlo44a and FCP5), representative sequences of the most common subtypes of the protist, and representative sequences from cold-blooded animals (represented as blue triangles and for *B. lapemi* sequences as rhomboids). Note that isolates from cold-blooded animals do not currently receive subtype designations. The analysis was conducted by a neighbor-joining method. Genetic distances were calculated using the Kimura two-parameter model. This analysis involved 79 nucleotide sequences, and there were a total of 2141 positions in the final dataset. The percentage of replicate trees in which the associated taxa clustered together in the bootstrap test (1000 replicates) are shown next to the branches. Bootstrap values lower than 75% are not displayed. *Proteromonas lacertae* was used as outgroup taxon to artificially root the tree. NHP means non-human primate.

In the NJ tree from the phylogenetic analysis that only included mammalian and avian subtypes, sequences from isolates 168, MFlo44a, FCP5, and Q52 formed a separate clade on a branch which includes ST3, ST4, ST8, ST10, ST16, and ST23 (Figure 1). Within the clade that included isolates 168, MFlo44a, FCP5, and Q52, it was observed that Q52 and FCP5 branched basally to 168 and MFlo44a. All branching within the clade formed by the new subtypes is supported by bootstrap values of between 83 and 100 (Figure 1). The topology of the NJ tree did not change in the phylogenetic analysis that included cold-blooded host sequences branched (Figure 2). In the tree that included sequences from cold-blooded hosts, the sequences from isolates 168, MFlo44a, FCP5, and Q52 still formed a separate clade that also included two sequences isolated from sea snakes (AY266471 and AY590115) that are identified in Figure 2 with blue rhomboids. These two sequences have previously been described as *B. lapemi*. The clade containing 168, MFlo44a, FCP5, Q52, and sea snake sequences is well-supported with a bootstrap value of 97. Within this clade, Q52 and FCP5 branch basally to a clade formed by 168 and MFlo44a, and the two *B. lapemi* sequences (Figure 2). Additionally, 168, MFlo44a, FCP5, and Q52 share a branch with avian and mammalian subtypes (ST3, ST4, ST16, ST8, ST10, ST16, and ST23) and additional sequences from other cold-blooded animals, including snakes, lizards, and frogs (Figure 2).

### 3.3. Pairwise Distance Comparisons

**Figure 3:**
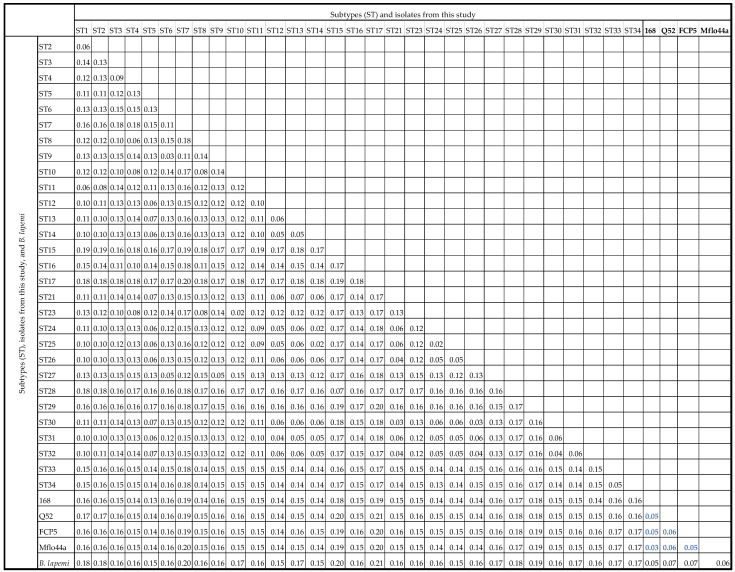
Pairwise distances between *Blastocystis* subtypes, isolates from this study, and *B. lapemi* (AY266471) full-length *ssu* rRNA gene sequences showing the average number of base substitutions per site. Analyses were conducted using the Kimura 2-parameter model and included 64 nucleotide sequences. There were a total of 2141 positions in the final dataset. Pairwise distance between isolates from this study are in bold blue font.

Pairwise distance comparisons were used to assess similarity between full-length *ssu* rRNA gene sequences of 168, MFlo44a, FCP5, and Q52 and other available full-length reference sequences of subtypes and reptilian, amphibian, and insect sequences. The sequence identity between 168, MFlo44a, FCP5, and Q52 and any known subtype ranged from 80–87% (Figure 3). The sequences of 168, MFlo44a, FCP5, and Q52 shared much more identity with each other than any previously described subtype with a sequence identity between 168, MFlo44a, FCP5, and Q52 ranging from 94–97% (Figure 3). Among the four full-length sequences described in this study, 168 and MFlo44a shared the most identity (97%) and Q52 the least (94–95%).

Pairwise distance comparisons were also performed between 168, MFlo44a, FCP5, and Q52 and full-length reference sequences of *B. lapemi*, as those sequences were the most similar sequences present in the GenBank database. The isolates 168, MFlo44a, FCP5, and Q52 shared more sequence similarity with *B. lapemi* than any subtype with an identity ranging from 93–95% (Figure 3). The sequence identity between 168, MFlo44a, FCP5, and Q52 and other reptilian, amphibian, and insect sequences were also assessed and found to range from 78–90%. Sequences from a python, a rhino iguana, and a leopard frog were among the most similar reptilian and amphibian sequences with an 86–90% similar sequence identity to 168, MFlo44a, FCP5, and Q52.

### 3.4. Designation of Blastocystis Subtypes ST35, ST36, ST37, and ST38

The analysis of full-length sequences from isolates 168, MFlo44a, FCP5, and Q52 in the present study supports their designation as new subtypes of *Blastocystis*. Thus, we propose the designation of these isolates as ST35-ST38. As 168 was reported as novel in 2018 and represents the first sequence identified in this clade, it is designated as ST35. Mflo44a and FCP5, which were first identified in 2020, are designated as ST36 and ST37. Q55 is the most recently identified of the four new subtypes and is designated as ST38.

## 4. Discussion

Interest in *Blastocystis* epidemiology and studies seeking to explore subtype diversity in new and more hosts are repainting the landscape of *Blastocystis* subtypes. In fact, of the 30 subtypes that are considered valid, 13 have been reported in the last five years [10,25,26,27]. The isolates in the present study were first described based on approximately 600 bp sequences of the *ssu* rRNA gene with the descriptions originating from three different studies from three different continents [18,19,20,22]. Based on the analysis of these sequences, it was observed in all cases that the most similar sequence available in the GenBank database was *B. lapemi*, which has only been observed in sea snakes of the genus *Lapemis* [21]. While the 600 bp sequences from these studies indicated that up to four new subtypes might be present, the lack of full-length *ssu* rRNA gene reference sequences and the confusion related to their similarity to a reptilian species of *Blastocystis* hindered their final classification as new subtypes. Here, we have obtained and analysed full-length *ssu* rRNA gene sequences for all four of these isolates to confirm their validity as new subtypes of *Blastocystis*.

The *Blastocystis* isolates in the present study came from a variety of mammalian species, including humans (168, ST35), and three different animal species, including a wild bat (*S. lilium*, MFlo44a, ST36), a wild rodent (*Heteromyidae*, FCP5, ST37), and a captive European water vole (*A. amphibius*, Q52, ST38). At the time of its initial description, ST35 was observed in two humans living in Brazil [18]. Subtype 36 was initially reported in two different animal hosts, the bat which is included in the present study (MFlo44a), and an identical sequence was also obtained from a gray four-eyed opossum (*Philander opossum*), both of which were identified in the state of Tabasco, Mexico [19]. Subtype 37 was identified in only a single host in its initial description, in a rodent from the state of Nayarit, Mexico [19]. Subtype 38 was also initially identified in two European water voles from the UK [20,22]. The hosts of the subtypes described in the present study were quite diverse and represent mammals with a variety of lifestyles and diets, ranging from omnivores to frugivores, as well as with a wide geographical range than includes three countries (Brazil, Mexico, and UK). The clade containing these new subtypes also includes *B. lapemi*, which originates from sea snakes from Singapore, a host which is a piscivore. Thus, there is no commonality within this clade based on host type, diet, or location.

Full-length *ssu* rRNA gene sequences generated in this study were compared to previously generated Sanger sequencing data for ST35-ST38. There was a high degree of agreement between sequences generated using both methods. However, a single mismatch was observed between all Sanger sequences and their corresponding Nanopore sequence. In all sequences, the mismatch was represented by the same base, with a C in the Sanger sequences being replaced with a T in the full-length sequences. This mismatch occurs in what would be the reverse primer region of the Sanger sequence, and as such, we hypothesize that the “true” sequence is best represented by the full-length sequence, which is not biased by the presence of a primer sequence across this region. Furthermore, the nearly 100% agreement between both sequencing methods supports the accuracy and validity of the full-length sequences used in the present study to describe ST35-ST38.

As the sequence in GenBank that is most similar to ST35-ST38 came from a reptile, we also investigated the phylogenetic relationship among accepted subtypes and select sequences from reptiles, amphibians, and insects. Currently subtypes are only assigned to isolates from mammalian and avian hosts [9]. However, even before the establishment of the subtyping system for mammalian and avian isolates, it was reported that isolates from amphibians and reptiles are closely related to isolates from humans and other endothermic hosts [28]. Regardless, the mingling of *Blastocystis* sequences of ecto- and endothermic origin leaves many open questions regarding the evolution and speciation of the organism. The most recent extensive exploration of this subject was conducted in 2016, and, at that time, only 17 subtypes had been described [29]. In the 2016 study, full-length *ssu* rRNA gene sequences were used to perform phylogenetic analyses of isolates from mammalian, avian, reptilian, amphibian, and insect hosts and concluded that *ssu* rRNA gene phylogenies and host origin do not correlate when sequences from across the spectra of potential *Blastocystis* hosts are considered. This same study did note the presence of three poikilothermic clades, which at the time, did not contain isolates from homeothermic animals. Notably, our analysis incorporating all currently identified subtypes indicates that two of these three clades now contain isolates from homeothermic animals. Another more recent study from 2017 used a subtyping system for poikilothermic sequences, which they designated as non-mammalian and avian STs (NMASTs); however, the NMAST system only used 246 bp for the analysis and classification [30]. Thus, the NMAST system is problematic in that it does not use the full-length *ssu* rRNA gene sequence for classification, as is shown to be essential for ascertaining novel STs and is the current recommendation in the field [1,25,29]. Given the seemingly close relationships between *Blastocystis* isolates from poikilothermic and homeothermic hosts, perhaps it is time to consider unifying the subtyping system to assist in clarifying the epidemiology of *Blastocystis* and in accurately naming new isolates, regardless of the host species.

## Data Availability

All relevant data are within the article. The sequences data were submitted to the GenBank database under the accession numbers OP720869-OP720872.
